# MSIsensor-RNA: Microsatellite Instability Detection for Bulk and Single-cell Gene Expression Data

**DOI:** 10.1093/gpbjnl/qzae004

**Published:** 2024-01-10

**Authors:** Peng Jia, Xuanhao Yang, Xiaofei Yang, Tingjie Wang, Yu Xu, Kai Ye

**Affiliations:** Department of Gynecology and Obstetrics, Center for Mathematical Medical, The First Affiliated Hospital of Xi’an Jiaotong University, Xi’an 710061, China; MOE Key Lab for Intelligent Networks & Networks Security, Faculty of Electronic and Information Engineering, Xi’an Jiaotong University, Xi’an 710049, China; School of Automation Science and Engineering, Faculty of Electronic and Information Engineering, Xi’an Jiaotong University, Xi’an 710049, China; MOE Key Lab for Intelligent Networks & Networks Security, Faculty of Electronic and Information Engineering, Xi’an Jiaotong University, Xi’an 710049, China; School of Automation Science and Engineering, Faculty of Electronic and Information Engineering, Xi’an Jiaotong University, Xi’an 710049, China; MOE Key Lab for Intelligent Networks & Networks Security, Faculty of Electronic and Information Engineering, Xi’an Jiaotong University, Xi’an 710049, China; School of Computer Science and Technology, Faculty of Electronic and Information Engineering, Xi’an Jiaotong University, Xi’an 710049, China; MOE Key Lab for Intelligent Networks & Networks Security, Faculty of Electronic and Information Engineering, Xi’an Jiaotong University, Xi’an 710049, China; Genome Institute, The First Affiliated Hospital of Xi’an Jiaotong University, Xi’an 710061, China; School of Life Science and Technology, Xi’an Jiaotong University, Xi’an 710049, China; Department of Gynecology and Obstetrics, Center for Mathematical Medical, The First Affiliated Hospital of Xi’an Jiaotong University, Xi’an 710061, China; MOE Key Lab for Intelligent Networks & Networks Security, Faculty of Electronic and Information Engineering, Xi’an Jiaotong University, Xi’an 710049, China; School of Automation Science and Engineering, Faculty of Electronic and Information Engineering, Xi’an Jiaotong University, Xi’an 710049, China; Genome Institute, The First Affiliated Hospital of Xi’an Jiaotong University, Xi’an 710061, China; School of Life Science and Technology, Xi’an Jiaotong University, Xi’an 710049, China

**Keywords:** Microsatellite instability, Gene expression, Single-cell RNA-seq, RNA-seq, Microarray

## Abstract

Microsatellite instability (MSI) is an indispensable biomarker in cancer immunotherapy. Currently, MSI scoring methods by high-throughput omics methods have gained popularity and demonstrated better performance than the gold standard method for MSI detection. However, the MSI detection method on expression data, especially single-cell expression data, is still lacking, limiting the scope of clinical application and prohibiting the investigation of MSI at a single-cell level. Herein, we developed MSIsensor-RNA, an accurate, robust, adaptable, and standalone software to detect MSI status based on expression values of MSI-associated genes. We demonstrated the favorable performance and promise of MSIsensor-RNA in both bulk and single-cell gene expression data in multiplatform technologies including RNA sequencing (RNA-seq), microarray, and single-cell RNA-seq. MSIsensor-RNA is a versatile, efficient, and robust method for MSI status detection from both bulk and single-cell gene expression data in clinical studies and applications. MSIsensor-RNA is available at https://github.com/xjtu-omics/msisensor-rna.

## Introduction

Microsatellite instability (MSI) refers to hypermutations of microsatellite sites due to inactivating alterations of mismatch repair (MMR) genes in malignancies [1,2]. Currently, MSI is an indispensable pan-cancer biomarker in cancer therapy and prognosis, and it is routinely examined in multiple cancer types, particularly in colorectal cancer (CRC), stomach adenocarcinoma (STAD), and uterine corpus endometrial carcinoma (UCEC) [2–5]. For example, MSI-positive patients are frequently resistant to 5-fluorouracil (5-FU) treatment but exhibit better responses to immune checkpoint blockade treatment [4,5].

In clinical settings, MSI detection mainly relies on the gold standard experimental method, MSI polymerase chain reaction (MSI-PCR) [6], which is laborious and time-consuming. With the advancement of next-generation sequencing technology, numerous features of genomics, epigenomics, transcriptomics, and histology are investigated, and novel MSI computational algorithms have been developed for a variety of scenarios [7–21]. Genomics-based methods quantify MSI according to genetic mutations at microsatellite sites, which achieve high accuracy and are becoming popular in clinical MSI detection. For example, MSIsensor [8] achieves a high concordance of 99.4% in detecting MSI on the Memorial Sloan Kettering-Integrated Mutation Profiling of Actionable Cancer Targets (MSK-IMPACT) panel [16]. Epigenomics-based method, MIRMMR, detects MSI using methylation levels in the MMR pathway with the area under the receiver operating characteristic curve (AUC) value of 0.97 [17]. In addition, transcription levels of MSI-associated genes exhibit correlations with MSI, hinting at the possibility of MSI detection using transcriptomics data [15,18,19]. Besides these high-throughput technologies, deep learning algorithms are also applied to hematoxylin and eosin (H&E)-stained slides to detect MSI [20,21]. However, all these MSI methods detect MSI at a sample level, lacking cell-level measurement of MSI. Recently, single-cell RNA sequencing (scRNA-seq) technology has enabled the investigation of cell-specific transcriptomes and shed light on tumor heterogeneity and tumor stages. In particular, the single-cell and spatial transcriptomes enable the dynamic analysis of MSI in the complex tumor microenvironment, such as in metastatic and recurrent cancer [22]. However, current MSI detection methods designed for bulk gene expression data do not perform well on scRNA-seq samples. For example, the only software for gene expression data, PreMSIm [18], provides fixed signatures and a fixed model for all cancers, which limits the wide application of the method. Moreover, the normalization method in PreMSIm also leads to poor performance with abnormal samples. Here, we developed MSIsensor-RNA, a robust method for MSI-associated gene detection and MSI evaluation for both bulk gene expression data and scRNA-seq data.

## Method

### Dataset

We downloaded RNA sequencing (RNA-seq) data of 1428 The Cancer Genome Atlas (TCGA) samples across CRC, STAD, and UCEC from TCGA Research Network (https://portal.gdc.cancer.gov) and obtained their MSI statuses as determined by gold standard (Table S1). We also obtained 141 RNA-seq samples from the International Cancer Genome Consortium (ICGC) Data Portal (https://dcc.icgc.org), and their MSI statuses were reported by MIMcall [23]. Another 106 RNA-seq samples with their matched MSI statuses were downloaded from the public publication of the Clinical Proteomic Tumor Analysis Consortium (CPTAC) [24]. We also downloaded microarray data and their MSI statuses of 1468 samples across CRC and STAD from the Gene Expression Omnibus (GEO) dataset (https://www.ncbi.nlm.nih.gov/geo). For scRNA-seq data, we got the gene expression data and their MSI statuses from 133 CRC samples in two recent publications [25,26].

### Overall design

**Figure 1:**
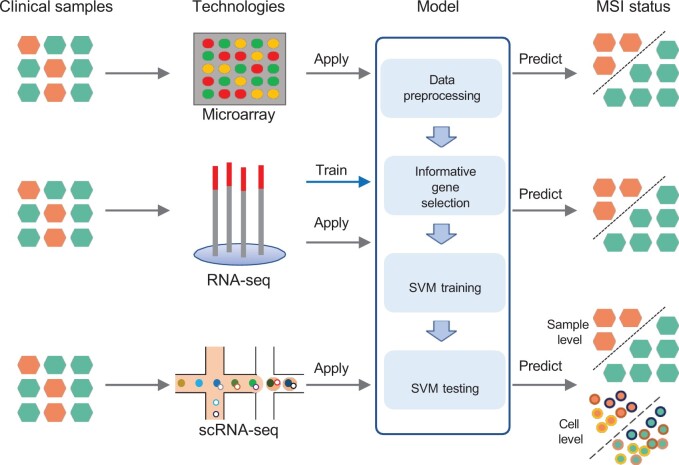
Workflow of MSIsensor-RNA MSIsensor-RNA includes four modules: data preprocessing, informative gene selection, SVM model training, and testing. MSIsensor-RNA selects informative genes and trains SVM model by RNA-seq samples from TCGA. MSI scores are predicted by the trained model for microarray, RNA-seq, and scRNA-seq samples. SVM, support vector machine; scRNA-seq, single-cell RNA-seq, RNA-seq, RNA sequencing; TCGA, The Cancer Genome Atlas; MSI, microsatellite instability.

The pipeline of MSIsensor-RNA consists of data preprocessing, informative gene selection, model training, and model testing (**Figure 1**, Figure S1). First, we preprocessed the expression values of samples from microarray, bulk RNA-seq, and scRNA-seq. Next, we selected an informative gene set for MSI detection from 1428 TCGA samples. Then, these TCGA samples were applied to train a machine learning model for MSI scoring. Finally, we applied the trained model to independent databases to test the performance of the MSIsensor-RNA for each cancer type.

### Data preprocessing

In MSIsensor-RNA, we accepted microarray expression value, fragments per kilobase million (FPKM), transcripts per million (TPM), and RNA-seq by expectation-maximization (RSEM) read count as input. All expression matrix values were increased by 1 and then subjected to a log_2_ transformation. Then, for each sample or cell, the expression values were normalized to follow a Gaussian distribution with a mean of 0 and a standard deviation of 1. For the scRNA-seq sample, to obtain accurate MSI status, we only included high-quality cells with at least 20% of genes detected for MSI detection. If the number of high-quality cells was less than 20, all cells were sorted by the ratio of detected genes in descending order, and the top 20 cells would be utilized for MSI detection. To solve the dropout problem of scRNA-seq, zero value was imputed by the average of the gene expression values in the given sample.

### Informative gene selection

We selected informative genes for MSI classification in terms of stability, discrimination, and generalization, utilizing a dataset of 1428 TCGA samples with Z-score-transformed gene expression values. Firstly, we removed ribosomal genes, mitochondrial genes, and genes with low FPKMs in TCGA dataset. Secondly, we selected genes with discriminative gene expression signatures between MSI samples and microsatellite stable (MSS) samples. We performed rank-sum test for expression values between MSI samples and MSS samples for each gene, and only genes with *P* < 0.01 (two-sided Wilcoxon rank-sum test) were included for the following analysis. Furthermore, we computed the log_2_ fold change of the *i*-th gene as in Equation 1.

(1) $$F^{i}=\left|\mathrm{lo}g_{2}\;\left(\frac{\frac{1}{n}{\sum_{j=1}^{n}G}_{j}^{i}}{\frac{1}{m-n}\;\sum_{k=n+1}^{m}G_{k}^{i}}\right)\right|$$

where *m* is the sample number for informative gene selection, *n* is the MSI sample number, and $G_{j}^{i}$ is the expression value of the *i*-th gene for the *j*-th sample. We only retained genes with log_2_ fold change > 0.5 for the next step. We next calculated the AUC for each gene, and only genes with AUC > 0.65 were regarded as candidate informative genes. To evaluate the generalization ability of the candidate informative genes, we employed both support vector machine (SVM) and random forest (RF) algorithms for each gene and calculated their respective 10-fold cross-validation scores. We then obtained two sorted gene lists based on the cross-validation results. Finally, we included the genes in the top 25% of both gene lists in the final informative gene set (Figure S2).

### Machine learning model training and testing

We built a SVM model to classify the MSI status for CRC, STAD, and UCEC in TCGA dataset. Firstly, we utilized SOMTE [27] to correct the imbalance between MSI and MSS in each cancer type by amplifying the MSI samples. Then, we utilized the expression values from the correct data as input to train the SVM model for MSI classification. To evaluate the performance of MSIsensor-RNA, we tested the trained model with 1848 independent samples from multiple platforms, including 247 RNA-seq, 1468 microarray, and 133 scRNA-seq samples. For a scRNA-seq sample, we calculated the MSI score with the SVM model for each high-quality cell. Then the average cell MSI score was used to evaluate the MSI status of a scRNA-seq sample.

### PreMSIm running

To compare the performance of MSIsensor-RNA with the only standalone software, PreMSIm, we also applied the data of microarray, RNA-seq, and scRNA-seq from 1848 independent samples to PreMSIm. For microarray and RNA-seq samples, we tested PreMSIm with two modes: PreMSIm-all and PreMSIm-split. In PreMSIm-all mode, we integrated all input samples from different databases and executed PreMSIm normalized and predicted modules. Conversely, the PreMSIm-split mode involved running PreMSIm separately for each database.

### Performance comparison of MSIsensor-RNA and PreMSIm

In MSIsensor-RNA, the predicted MSI probability by the SVM model was used to score the MSI status. The probabilities were further transformed into MSI statuses by the Youden index [28]. We first compared the MSIsensor-RNA score between MSI and MSS samples to test the performance of MSIsensor-RNA in multiple platforms using a rank-sum test. To further evaluate the performance of both two MSI detection methods, we calculated AUC, accuracy, F1-score, precision, sensitivity, and specificity of MSIsensor-RNA and PreMSIm across different sequencing platforms.

### Robustness testing of MSIsensor-RNA and PreMSIm

To test the performance of MSIsensor-RNA and PreMSIm at different normalization methods, we tested these two methods with FPKM, TPM, and RSEM read count formats of TCGA samples and calculated the AUC, F1-score, accuracy, precision, sensitivity, and specificity for each normalization method. To overcome the bias of different normalization methods and sequencing technologies, we normalized the input data of each sample to a Gaussian distribution with a mean of 0 and a standard deviation of 1. However, in PreMSIm, the normalization process was performed by genes, which means the normalized input data of a sample would be influenced by other samples in the bulk. Here, we tested the PreMSIm in two ways. Firstly, we input TCGA samples by three cancer types and calculated the performance of the predicted MSI statuses. Secondly, we input all TCGA samples together to evaluate their performance. We further compared the MSI results and performance of these two methods and found that the performance of PreMSIm was affected by the input way.

## Results and discussion

The workflow of MSIsensor-RNA includes four modules (Figure 1, Figure S1). First, we preprocessed the expression values of microarray, bulk RNA-seq, and scRNA-seq data. Then, we selected a set of informative genes for MSI detection. Next, we trained a SVM model to estimate MSI scores using the gene expression values of the selected informative genes. Finally, we applied the trained model to predict MSI scores for either one clinical sample or a single cell (Table S1). For a given scRNA-seq sample, we also developed a model to report the MSI status of this sample by integrating MSI scores of cells within.

We first preprocessed all gene expression data and then applied the gene selection module to 1428 samples (the Z-score-transferred gene expression values) from three MSI-popular cancer types (CRC, STAD, and UCEC) in TCGA dataset. Finally, we obtained 109 informative genes for MSI classification. We also performed this step for each type of CRC, STAD, and UCEC, yielding 397, 206, and 86 informative genes, respectively (Figures S3 and S4; Tables S2–S5). We found that only eight informative genes were detected in all three cancer types. Among these genes, we found that *MLH1* was the most important informative gene for MSI detection, as confirmed by previous reports [15,18,19] (Figure S5).

To assess the performance of MSIsensor-RNA in bulk sample data, we first trained tumor-specific models for CRC, STAD, and UCEC, as well as a model for all three MSI-popular cancer types in TCGA dataset. Then, we compared the two kinds of models (tumor-specific and MSI-popular) with the standalone software, PreMSIm, in terms of the AUC, accuracy, sensitivity, and specificity in 1715 independent samples (1468 microarray samples and 247 bulk RNA-seq samples). Notably, MSIsensor-RNA normalizes the expression value of informative genes for each sample independently, while PreMSIm must normalize each gene for multiple samples at the same time. Thus, we examined PreMSIm using both PreMSIm-all and PreMSIm-split modes (see Method for details).

**Figure 2:**
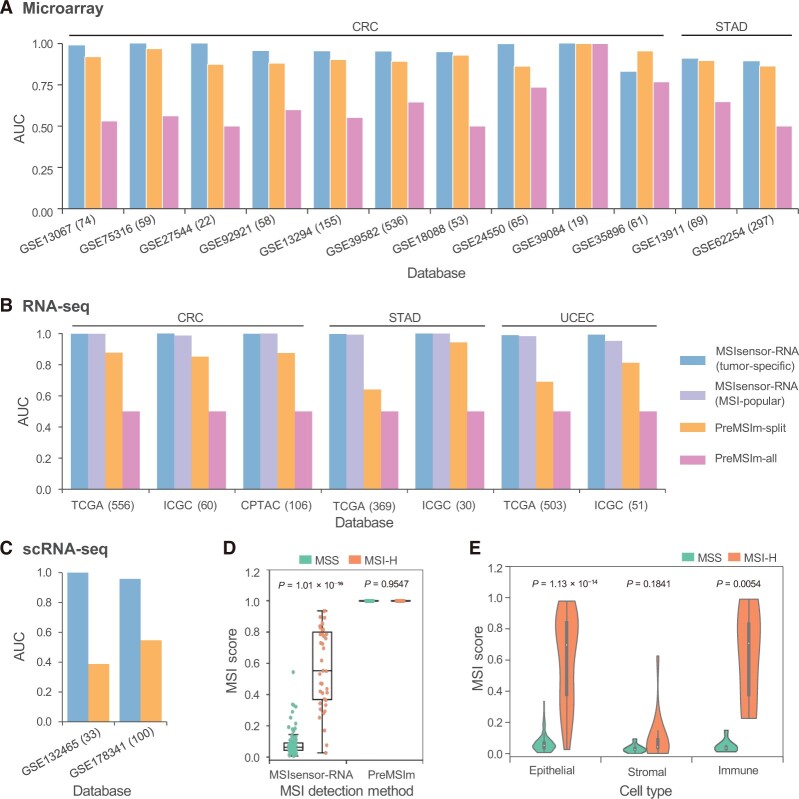
Performance of MSIsensor-RNA AUC of MSIsensor-RNA and PreMSIm in microarray (**A**), RNA-seq (**B**), and scRNA-seq (**C**) samples. In PreMSIm-all mode, all input samples from different databases are integrated for PreMSIm running. Conversely, the PreMSIm-split mode involves running PreMSIm separately for each database. **D**. Boxplot of MSI scores in scRNA-seq samples. **E**. Violin plot of MSI scores of different cell types in scRNA-seq samples. Epithelial, stromal, and immune cell types were defined in the previous study [25]. *P* values were obtained through a two-sided rank-sum test. Tumor-specific indicates MSI results with the tumor-specific model; MSI-popular indicates MSI results with three MSI-popular cancer types. AUC, the area under the receiver operating characteristic curve; CRC, colorectal cancer; STAD, stomach adenocarcinoma; UCEC, uterine corpus endometrial carcinoma; ICGC, International Cancer Genome Consortium; CPTAC, Clinical Proteomic Tumor Analysis Consortium; MSS, microsatellite stable; MSI-H, microsatellite instability high.

For microarray data, we computed MSI status by MSIsensor-RNA and PreMSIm in 1468 samples from 12 GEO accessions. The result showed that MSIsensor-RNA predicted MSI with an AUC value of 0.952, while PreMSIm only obtained an AUC value of 0.628 in PreMSIm-split mode and an AUC value of 0.912 in PreMSIm-all mode (**Figure 2**A, Figures S6 and S7; Tables S6 and S7). Meanwhile, MSIsensor-RNA achieved much higher sensitivity than PreMSIm-split and PreMSIm-all (MSIsensor-RNA: 0.968; PreMSIm-split: 0.912; PreMSIm-all: 0.384), and performed comparable specificity with PreMSIm-split and PreMSIm-all (MSIsensor-RNA: 0.843; PreMSIm-split: 0.912; PreMSIm-all: 0.873).

To evaluate the performance using bulk RNA-seq data, we compared MSIsensor-RNA and two modes of PreMSIm on 247 independent samples from ICGC and CPTAC. We noticed that MSIsensor-RNA achieved an AUC value of 0.997 in the tumor-specific model and an AUC value of 0.985 in the MSI-popular model, which were significantly greater than PreMSIm-all (AUC = 0.500) and PreMSIm-split (AUC = 0.870) (Figure 2B, Figures S8 and S9; Tables S8 and S9). In addition, MSIsensor-RNA performed much better than PreMSIm for both sensitivity (MSIsensor-RNA with tumor-specific model: 0.951; MSIsensor-RNA with MSI-popular model: 0.973; PreMSIm-split: 0.834; PreMSIm-all: 0.250) and specificity (MSIsensor-RNA with tumor-specific mode: 1.000; MSIsensor-RNA with MSI-popular model: 0.923; PreMSIm-split: 0.906; PreMSIm-all: 0.750). To further investigate the robustness of MSIsensor-RNA for different input data types, we evaluated the performance of MSIsensor-RNA and PreMSIm with FPKM, read count, and TPM normalized samples in TCGA as input. We found that MSIsensor-RNA achieved AUC = 0.982 ± 0.040, indicating its robustness regardless of the measurement methods of gene expression (Table S10).

To assess the performance of MSIsensor-RNA and PreMSIm in scRNA-seq samples, we applied the trained model from TCGA dataset to 23,902 high-quality cells from 133 samples to obtain sample-specific MSI status and compared it to the ratio of cells labeled as MSI by PreMSIm. The result showed that MSIsensor-RNA detected MSI for scRNA-seq samples with AUC = 0.9583, sensitivity = 0.9231, and specificity = 0.9362, while PreMSIm had AUC = 0.4969, sensitivity = 1.0000, and specificity = 0.0319 (Figure 2C, Figure S10; Tables S11 and S12). The sample-level MSI scores based on scRNA-seq were significantly different between MSI and MSS samples detected by MSIsensor-RNA (two-sided rank-sum test, *P* = 1.01 × 10^−16^), while no significant difference was detected by PreMSIm (two-sided rank-sum test, *P* = 0.9547) (Figure 2D). Having established the effectiveness of MSIsensor-RNA on scRNA-seq samples, we investigated cell-level MSI. We computed the MSI scores of 21,438 high-quality cells from 100 samples (GSE178341) and found that MSI score was correlated with cell type. For example, MSI scores of epithelial and immune cells in MSI samples were greater than those in MSS samples, while no significant difference was detected between MSI and MSS for stromal cells (Figure 2E, Figure S11; Table S13). This indicates the potential of MSIsensor-RNA to assess MSI at the single-cell level, providing a novel measurement for the investigation of tumorigenic processes.

MSI is important for the prognosis assessment of both 5-FU chemotherapy [4] and immunotherapy [5]. In addition to gold standard experimental methods [6], MSI status is also evaluated according to genomic sequencing data [7–14], gene expression data [15,18,19], methylation data [17], and H&E-stained slides [20,21]. Compared to variants in microsatellite regions, gene expression values are more directly reflective of the features of MSI and easier to obtain. In this study, we developed a robust method, MSIsensor-RNA, for MSI detection with gene expression data. MSIsensor-RNA provides informative gene selection, model training, and MSI detection modules and is able to process data from multiple platforms, including microarray, RNA-seq, and scRNA-seq. Compared to the standalone method PreMSIm, MSIsensor-RNA also provides modules for informative gene selection and model training, enabling users to apply it across different cancer types. MSIsensor-RNA also improves the normalization method of the data, yielding a more robust result than PreMSIm (Figure 2). In addition, MSIsensor-RNA facilitates the evaluation of MSI status at the single-cell level, which will be critical to better understanding the mechanism of MSI in cancer immunotherapy in the future.

In most MSI detection methods, such as MSIsensor [10] and MSIsensor-pro [11], MSI is quantified according to genetic mutations at microsatellite sites, the consequence of MSI, rather than the deficiency of the MMR system, the direct cause of MSI. In this study, a set of MSI-associated genes was identified, and their expression values were used for MSI evaluation. We found that *MLH1* is the most important gene in all tested cancer types. In addition, unexpected expression of *MLH1* is commonly observed in Lynch syndrome [29]. Thus, we tested the performance of MSIsensor-RNA for samples with abnormal *MLH1* expression. We trained a model based on all informative genes and tested it using samples with simulated abnormal *MLH1* gene expression (Table S14). We found that the model achieved AUC values of 0.974 and 0.972 when we set the *MLH1* expression value as the maximum and minimum of all gene expression values, respectively. Furthermore, when *MLH1* was excluded from the informative gene set, MSIsensor-RNA also achieved an AUC value of 0.977, indicating the robustness of MSIsensor-RNA for MSI detection.

We demonstrated that MSIsensor-RNA achieved higher performance than other methods based on gene expression and comparable performance compared to DNA-based methods (Table S15). In our study design, MSIsensor-RNA detects MSI according to the gene expression signatures of genes on MSI-associated pathways, while MSIsensor evaluates MSI by computing the ratio of somatic microsatellite mutations. Although MSIsensor achieved slightly higher performance than MSIsensor-RNA, it cannot replace the applications of MSIsensor-RNA in gene expression data. Currently, MSIsensor-RNA reports favorable performance in all three MSI-popular cancers, including CRC, STAD, and UCEC. The MSI features vary across different cancer types. Thus, the model obtained low performance when the testing samples were inconsistent with training samples in cancer types (Tables S16–S18). Therefore, the performance of MSIsensor-RNA in other cancer types needs further validation in the future. Another characteristic of MSIsensor-RNA is that its performance is not stable when trained with only a small number of positive samples. For example, in gastric cancer, the MSI high (MSI-H) training samples in stages I and IV are much fewer compared to other stages, leading to lower performance in these two stages (Tables S19 and S20).

## Conclusion

MSIsensor-RNA is a cross-platform, efficient, and robust method for MSI status determination from both bulk and single-cell gene expression data. We demonstrate the effectiveness and robustness of MSIsensor-RNA across different platforms, hinting at its potential in clinical research. Moreover, MSIsensor-RNA enables single cell-level MSI evaluation, providing a new tool to discover the role of MSI in the tumorigenic process and to monitor cell-level dynamic changes during immunotherapy.

## Code availability

MSIsensor-RNA is available at https://ngdc.cncb.ac.cn/biocode/tool/BT7385 and https://github.com/xjtu-omics/msisensor-rna.

## CRediT author statement

**Peng Jia:** Methodology, Software, Validation, Formal analysis, Investigation, Visualization, Writing – original draft, Writing – review & editing. **Xuanhao Yang:** Methodology, Software, Formal analysis, Investigation, Data curation, Writing – original draft. **Xiaofei Yang:** Methodology, Funding acquisition. **Tingjie Wang:** Methodology. **Yu Xu:** Methodology. **Kai Ye:** Methodology, Resources, Conceptualization, Supervision, Project administration, Funding acquisition. All authors have read and approved the final manuscript.

## Supplementary material

Supplementary material is available at *Genomics, Proteomics & Bioinformatics* online (https://doi.org/10.1093/gpbjnl/qzae004).

## Competing interests

The authors have declared no competing interests.

## Supplementary Material

qzae004_Supplementary_Data
