# Supplementary material for: MSIsensor-RNA: Microsatellite Instability Detection for Bulk and Single-cell Gene Expression Data
Source: Genomics Proteomics Bioinformatics. 2024 Jan 10;22(3):qzae004. doi: 10.1093/gpbjnl/qzae004 (PMC12016039; doi:10.1093/gpbjnl/qzae004)
Supplement: qzae004_Supplementary_Data [file qzae004_supplementary_data.zip › Figure S7.pptx]

## Slide 1
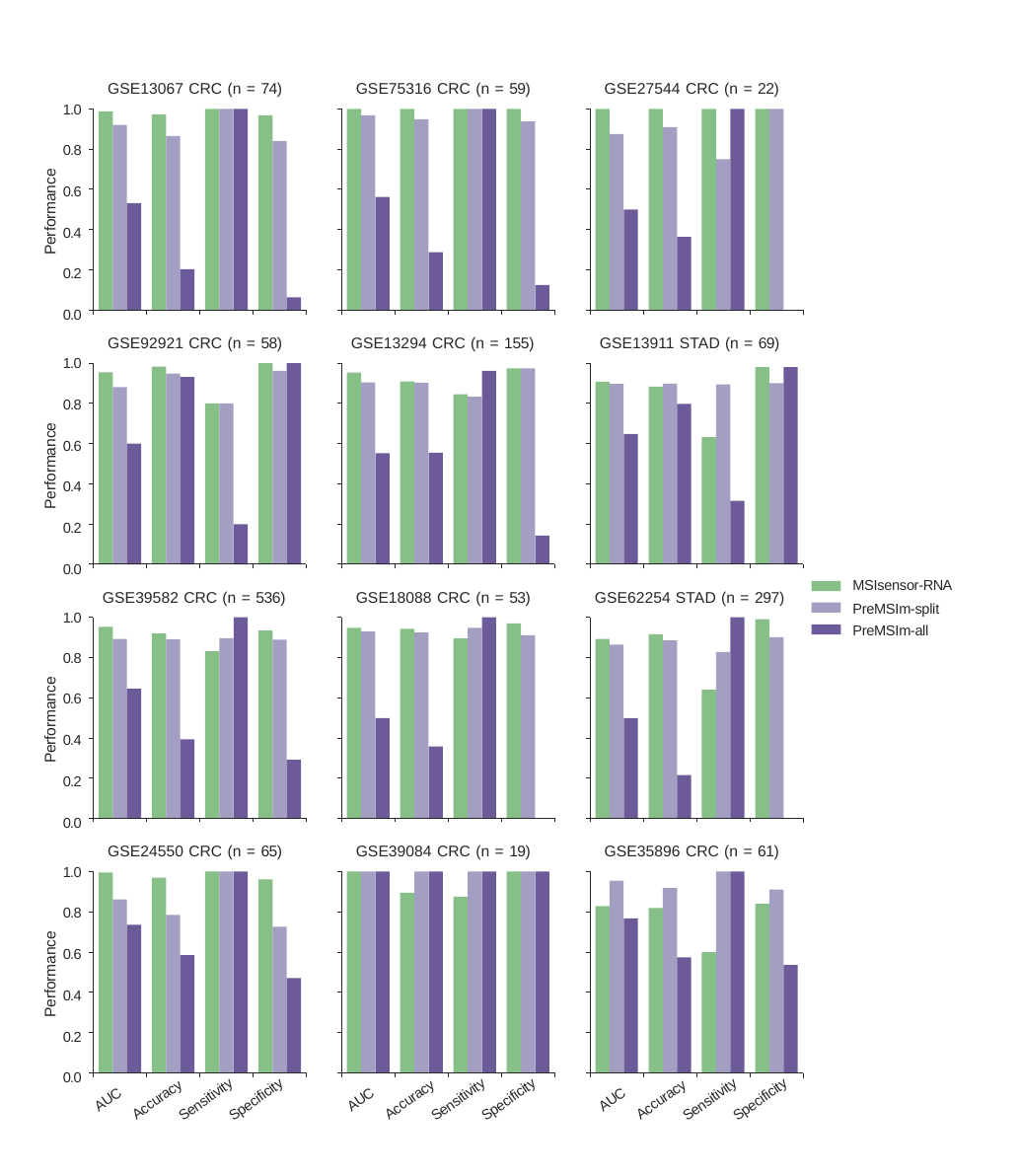

GSE13067 CRC (n = 74)
GSE75316 CRC (n = 59)
GSE27544 CRC (n = 22)
1.0
0.8
0.6
0.4
0.2
0.0
Performance
GSE92921 CRC (n = 58)
GSE13294 CRC (n = 155)
GSE13911 STAD (n = 69)
1.0
0.8
0.6
0.4
0.2
0.0
Performance
MSIsensor-RNA
PreMSIm-split PreMSIm-all
GSE39582 CRC (n = 536)
GSE18088 CRC (n = 53)
GSE62254 STAD (n = 297)
1.0
0.8
0.6
0.4
0.2
0.0
Performance
GSE24550 CRC (n = 65)
GSE39084 CRC (n = 19)
GSE35896 CRC (n = 61)
1.0
0.8
0.6
0.4
0.2
0.0
Performance
AUC
AUC
AUC
Accuracy
Accuracy
Specificity
Specificity
Sensitivity
Sensitivity
Accuracy
Specificity
Sensitivity
