# Supplementary material for: MSIsensor-RNA: Microsatellite Instability Detection for Bulk and Single-cell Gene Expression Data
Source: Genomics Proteomics Bioinformatics. 2024 Jan 10;22(3):qzae004. doi: 10.1093/gpbjnl/qzae004 (PMC12016039; doi:10.1093/gpbjnl/qzae004)
Supplement: qzae004_Supplementary_Data [file qzae004_supplementary_data.zip › Figure S9.pptx]

## Slide 1
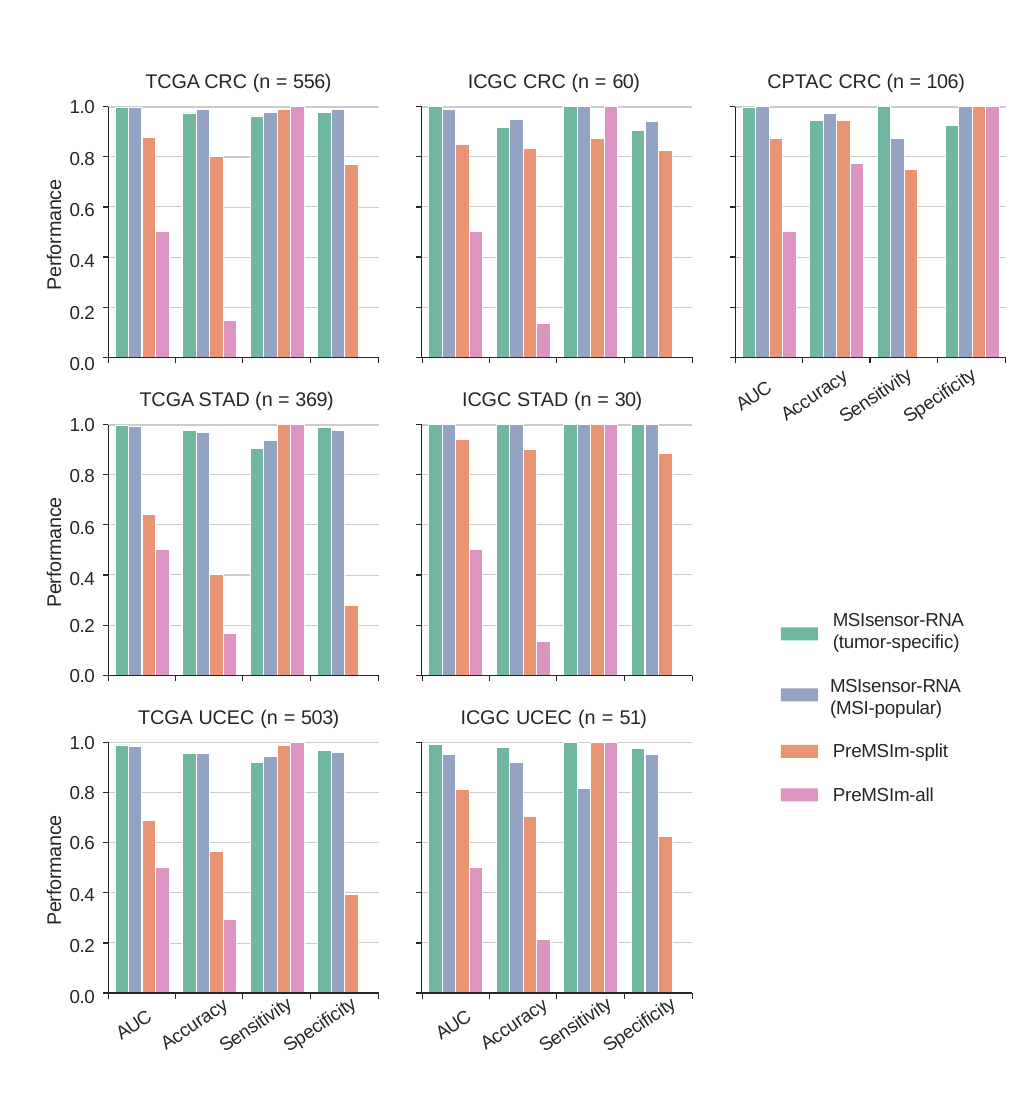

TCGA CRC (n = 556)
ICGC CRC (n = 60)
CPTAC CRC (n = 106)
1.0
0.8
0.6
0.4
0.2
0.0
Performance
TCGA STAD (n = 369)
ICGC STAD (n = 30)
AUC
Accuracy
Specificity
Sensitivity
1.0
0.8
0.6
0.4
Performance
MSIsensor-RNA (tumor-specific)
0.2
0.0
MSIsensor-RNA (MSI-popular)
TCGA UCEC (n = 503)
ICGC UCEC (n = 51)
1.0
PreMSIm-split
0.8
PreMSIm-all
Performance
0.6
0.4
0.2
0.0
AUC
AUC
Accuracy
Accuracy
Specificity
Specificity
Sensitivity
Sensitivity
