# Supplementary material for: MSIsensor-RNA: Microsatellite Instability Detection for Bulk and Single-cell Gene Expression Data
Source: Genomics Proteomics Bioinformatics. 2024 Jan 10;22(3):qzae004. doi: 10.1093/gpbjnl/qzae004 (PMC12016039; doi:10.1093/gpbjnl/qzae004)
Supplement: qzae004_Supplementary_Data [file qzae004_supplementary_data.zip › Figure S10.pptx]

## Slide 1
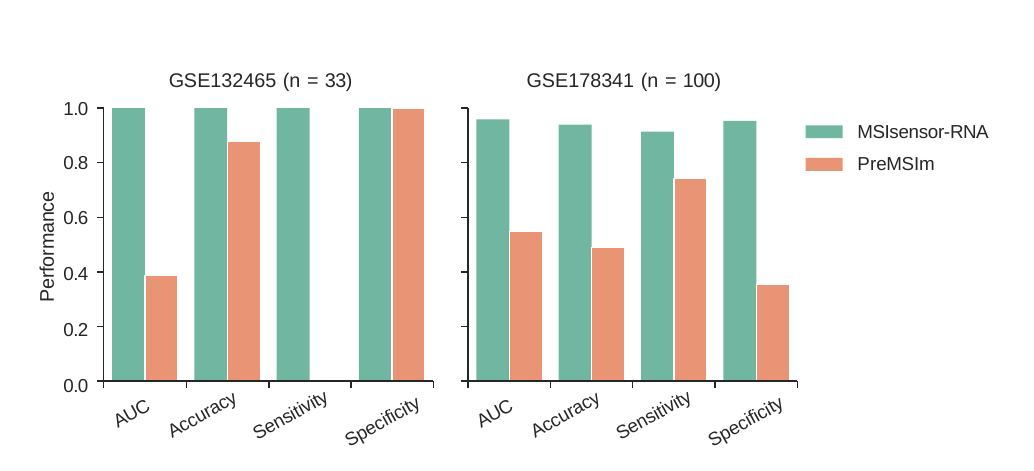

GSE132465 (n = 33)
GSE178341 (n = 100)
1.0
MSIsensor-RNA PreMSIm
0.8
Performance
0.6
0.4
0.2
0.0
AUC
AUC
Accuracy
Accuracy
Sensitivity
Sensitivity
Specificity
Specificity
