# Supplementary material for: MSIsensor-RNA: Microsatellite Instability Detection for Bulk and Single-cell Gene Expression Data
Source: Genomics Proteomics Bioinformatics. 2024 Jan 10;22(3):qzae004. doi: 10.1093/gpbjnl/qzae004 (PMC12016039; doi:10.1093/gpbjnl/qzae004)
Supplement: qzae004_Supplementary_Data [file qzae004_supplementary_data.zip › Supplementary material captions.docx]

# Supplementary material

**Figure S1 Workflow of training and testing for MSIsensor-pro**

The diagram illustrates the detailed steps of training and testing for MSIsensor-pro. WES, whole exome sequencing. The MSIsensor score indicates the MSI score calculated by MSIsensor. Meanwhile, the MSIsensor-RNA score represents the MSI score calculated by MSIsensor-RNA.

**Figure S2 Workflow of informative gene selection**

MSIsensor-RNA selects informative genes based on criteria such as stability, discrimination, and generalization. Initially, mitochondrial genes and ribosomal genes are excluded from consideration. Subsequently, genes with expression values that do not exhibit significant differences between MSI and MSS samples (as determined by a two-sided Wilcoxon rank-sum test) are filtered out. Finally, genes with expression values that demonstrate a high generalization score for MSI detection are retained.

**Figure S3 Venn plot showing the intersection of the informative genes across different cancer types**

The pink circle represents the informative genes generated from samples of three MSI-popular cancer types including CRC, STAD, and UCEC.

**Figure S4 Upset plot showing the intersection of the informative genes across this study and the other three publications**

All four studies utilized *MLH1* as an informative gene.

**Figure S5 Violin plot of expression values of mismatch repair genes in MSI and MSS samples**

A two-sided Wilcoxon rank-sum test is implemented to compare MSI scores between MSI and MSS samples. FPKM, fragments per kilobase million; ns, not significant; *, *P* < 0.0001.

**Figure S6 Violin plot of MSI score by MSIsensor-RNA in microarray data**

The violin plot shows that MSI scores by MSIsensor-RNA in MSI are significantly greater than those in MSS samples. A two-sided Wilcoxon rank-sum test is implemented to compare MSI scores between MSI and MSS samples. ns, not significant; *, *P* < 0.05; **, P < 0.01; ***, P < 0.001; ****, *P* < 0.0001.

**Figure S7 Performance of MSIsensor-RNA, PreMSIm-all, and PreMSIm-split for microarray data**

The bar plot shows that MSIsensor-RNA outperforms PreMSIm-all and PreMSIm-split across AUC, accuracy, sensitivity, and specificity. In PreMSIm-all mode, all input samples from different databases are integrated for PreMSIm running. Conversely, the PreMSIm-split mode involves running PreMSIm separately for each database.

**Figure S8 Violin plot of MSI score by MSIsensor-RNA in bulk RNA-seq data**

The violin plot shows that MSI scores by MSIsensor-RNA in MSI are significantly greater than those in MSS samples. A two-sided Wilcoxon rank-sum test is implemented to compare MSI scores between MSI and MSS samples.

**Figure S9 Performance of MSIsensor-RNA, PreMSIm-all, and PreMSIm-split for bulk RNA-seq data**

The bar plot shows that MSIsensor-RNA outperforms PreMSIm-all and PreMSIm-split across AUC, accuracy, sensitivity, and specificity in all three databases. PreMSIm performs nearly 100% sensitivities but about 50% specificities in TCGA and ICGC. Meanwhile, PreMSIm predicts MSI status with 100% specificities but 0.7 sensitivities in PreMSIm-split and 0 in PreMSIm-all in CPTAC dataset.

**Figure S10 Performance of MSIsensor-RNA, PreMSIm-all, and PreMSIm-split for scRNA-seq data**

The bar plot shows that MSIsensor-RNA outperforms PreMSIm-all and PreMSIm-split across AUC, accuracy, sensitivity, and specificity in the two databases.

**Figure S11 Scatter plot shows the distribution of MSI score and cell ratio of differential cell types in GSE178341**

The result shows that epithelial cell is a large proportion of tumor samples, while stromal cell and immune cell are only less than 25%. The MSI scores of epithelial cells and immune cells in MSI samples are significantly greater than those in MSS samples, but MSI scores in stromal cells have no significant difference between MSI and MSS samples.

**Table S1 Overview of samples in this study**

**Table S2 Details of informative genes in CRC**

**Table S3 Details of informative genes in** **STAD**

**Table S4 Details of informative genes in** **UCEC**

**Table S5 Details of informative genes in three MSI-popular cancers**

**Table S6 MSI results of microarray samples by MSIsensor-RNA and PreMSIm**

**Table S7 MSI detection performance of MSIsensor-RNA and PreMSIm in microarray samples**

**Table S8 MSI results of RNA-seq samples by MSIsensor-RNA and PreMSIm**

**Table S9 MSI detection performance of MSIsensor-RNA and PreMSIm in RNA-seq samples**

**Table S10 MSI detection performance of MSIsensor-RNA and PreMSIm in different normalized samples**

**Table S11 MSI results of scRNA-seq samples by MSIsensor-RNA**

**Table S12 MSI detection performance of MSIsensor-RNA and preMSIm in scRNA-seq samples**

**Table S13 MSI results of scRNA-seq cells by MSIsensor-RNA**

**Table S14 Performance of MSIsensor-RNA with abnormal *MLH1* expression values**

**Table S15 Performance of MSIsensor-RNA and MSIsensor in TCGA dataset**

**Table S16 AUC of MSIsensor-RNA with inconsistent training and testing samples**

**Table S17 Performance of train models for cancer with low-frequency MSI**

**Table S18 Performance of MSIsensor-RNA for cancer with low-frequency MSI by 5-fold cross-validation**

**Table S19 Performance of MSIsensor-RNA across different cancer stages**

**Table S20 Summary of MSI-H training samples for STAD in Table S19**
