# Supplementary material for: MSIsensor-RNA: Microsatellite Instability Detection for Bulk and Single-cell Gene Expression Data
Source: Genomics Proteomics Bioinformatics. 2024 Jan 10;22(3):qzae004. doi: 10.1093/gpbjnl/qzae004 (PMC12016039; doi:10.1093/gpbjnl/qzae004)
Supplement: qzae004_Supplementary_Data [file qzae004_supplementary_data.zip › Table S10-done.docx]

**Table S10 MSI detection performance of MSIsensor-RNA and PreMSIm in different normalized samples**

| **MSI detected Method** | **AUC** | **F1-score** | **Accuracy** | **Precision** | **Sensitivity** | **Specificity** | **Normalization format** |
| --- | --- | --- | --- | --- | --- | --- | --- |
| MSIsensor-RNA | 0.9773 | 0.9268 | 0.9760 | 0.9383 | 0.9157 | 0.9880 | gene_expected_count |
| MSIsensor-RNA | 0.9863 | 0.9329 | 0.9780 | 0.9444 | 0.9217 | 0.9892 | RSEM_gene_fpkm |
| MSIsensor-RNA | 0.9862 | 0.9329 | 0.9780 | 0.9444 | 0.9217 | 0.9892 | RSEM_gene_tpm |
| MSIsensor-RNA | 0.9773 | 0.9268 | 0.9760 | 0.9383 | 0.9157 | 0.9880 | RSEM_Hugo_norm_count |
| PreMSIm | 0.8971 | 0.8421 | 0.9491 | 0.8662 | 0.8193 | 0.9749 | gene_expected_count |
| PreMSIm | 0.9151 | 0.8632 | 0.9550 | 0.8712 | 0.8554 | 0.9749 | RSEM_gene_fpkm |
| PreMSIm | 0.8917 | 0.8193 | 0.9401 | 0.8193 | 0.8193 | 0.9641 | RSEM_gene_tpm |
| PreMSIm | 0.9386 | 0.8976 | 0.9660 | 0.8976 | 0.8976 | 0.9796 | RSEM_Hugo_norm_count |

*Note*: The data was downloaded from <https://xenabrowser.net/datapages.>TPM/tpm, transcripts per million; FPKM/fpkm, fragments per kilobase million; RSEM, RNA-seq by expectation-maximization.
