# Supplementary material for: MSIsensor-RNA: Microsatellite Instability Detection for Bulk and Single-cell Gene Expression Data
Source: Genomics Proteomics Bioinformatics. 2024 Jan 10;22(3):qzae004. doi: 10.1093/gpbjnl/qzae004 (PMC12016039; doi:10.1093/gpbjnl/qzae004)
Supplement: qzae004_Supplementary_Data [file qzae004_supplementary_data.zip › Table S12-done.docx]

**Table S12 MSI detection performance of MSIsensor-RNA and preMSIm in scRNA-seq samples**

| **Database** | **Cancer type** | **No. of samples** | **MSI detected method** | **AUC** | **F1-score** | **Accuracy** | **Sensitivity** | **Specificity** | **Precision** |
| --- | --- | --- | --- | --- | --- | --- | --- | --- | --- |
| GSE132465 | CRC | 33 | MSIsensor-RNA | 1.0000 | 1.0000 | 1.0000 | 1.0000 | 1.0000 | 1.0000 |
| GSE132465 | CRC | 33 | PreMSIm | 0.3879 | 0.0000 | 0.8788 | 0.0000 | 1.0000 | NA |
| GSE178341 | CRC | 100 | MSIsensor-RNA | 0.9591 | 0.9143 | 0.9400 | 0.9143 | 0.9538 | 0.9143 |
| GSE178341 | CRC | 100 | PreMSIm | 0.5473 | 0.5049 | 0.4900 | 0.7429 | 0.3538 | 0.3824 |
| All | CRC | 133 | MSIsensor-RNA | 0.9583 | 0.8889 | 0.9323 | 0.9231 | 0.9362 | 0.8571 |
| All | CRC | 133 | PreMSIm | 0.4969 | 0.4615 | 0.3158 | 1.0000 | 0.0319 | 0.3000 |

*Note*: NA: not available; CRC colorectal cancer.
