# Supplementary material for: MSIsensor-RNA: Microsatellite Instability Detection for Bulk and Single-cell Gene Expression Data
Source: Genomics Proteomics Bioinformatics. 2024 Jan 10;22(3):qzae004. doi: 10.1093/gpbjnl/qzae004 (PMC12016039; doi:10.1093/gpbjnl/qzae004)
Supplement: qzae004_Supplementary_Data [file qzae004_supplementary_data.zip › Table S14-done.docx]

**Table S14 Performance of MSIsensor-RNA with abnormal *MLH1* expression values**

| **Genes in train data (TCGA)** | **Genes in test data (ICGC)** | **AUC** |
| --- | --- | --- |
| All informative genes | All informative genes | 0.982 |
| All informative genes | Set *MLH1* expression as maximum value of all samples | 0.974 |
| All informative genes | Set *MLH1* expression as 0 | 0.972 |
| Informative genes excluded *MLH1* | Informative genes excluded *MLH1* | 0.977 |
