# Supplementary material for: MSIsensor-RNA: Microsatellite Instability Detection for Bulk and Single-cell Gene Expression Data
Source: Genomics Proteomics Bioinformatics. 2024 Jan 10;22(3):qzae004. doi: 10.1093/gpbjnl/qzae004 (PMC12016039; doi:10.1093/gpbjnl/qzae004)
Supplement: qzae004_Supplementary_Data [file qzae004_supplementary_data.zip › Table S15-done.docx]

**Table S15 Performance of MSIsensor-RNA and MSIsensor in TCGA dataset**

| **Method** | **No. of samples** | **Cancer type** | **AUC** | **F1-score** | **Accuracy** | **Sensitivity** | **Specificity** | **Precision** |
| --- | --- | --- | --- | --- | --- | --- | --- | --- |
| MSIsensor-RNA | 483 | CRC | 0.9736 | 0.8382 | 0.9545 | 0.9735 | 0.8382 | 0.8382 |
| MSIsensor | 483 | CRC | 0.9870 | 0.8667 | 0.9586 | 0.9590 | 0.9559 | 0.7927 |
| MSIsensor-RNA | 298 | STAD | 0.9777 | 0.8911 | 0.9631 | 0.9918 | 0.8333 | 0.9574 |
| MSIsensor | 298 | STAD | 1.0000 | 0.9515 | 0.9832 | 1.0000 | 0.9074 | 1.0000 |
| MSIsensor-RNA | 428 | UCEC | 0.9485 | 0.8235 | 0.8879 | 0.9116 | 0.8358 | 0.8116 |
| MSIsensor | 428 | UCEC | 0.9853 | 0.9421 | 0.9650 | 0.9898 | 0.9104 | 0.9760 |
| MSIsensor-RNA | 1209 | Three MSI-popular cancer types | 0.9557 | 0.8204 | 0.9214 | 0.9412 | 0.8477 | 0.7949 |
| MSIsensor | 1209 | Three MSI-popular cancer types | 0.9881 | 0.9219 | 0.9669 | 0.9790 | 0.9219 | 0.9219 |

*Note*: 70% samples in TCGA are used to train a model for MSIsensor-RNA, and the remain 30% TCGA samples are uesed to compare the performance between MSIsensor and MSIsensor-RNA; NA: not available; CRC colorectal cancer; STAD, stomach adenocarcinoma; UCEC, uterine corpus endometrial carcinoma; AUC, area under the receiver operating characteristic curve.
