# Supplementary material for: MSIsensor-RNA: Microsatellite Instability Detection for Bulk and Single-cell Gene Expression Data
Source: Genomics Proteomics Bioinformatics. 2024 Jan 10;22(3):qzae004. doi: 10.1093/gpbjnl/qzae004 (PMC12016039; doi:10.1093/gpbjnl/qzae004)
Supplement: qzae004_Supplementary_Data [file qzae004_supplementary_data.zip › Table S16-done.docx]

**Table S16 AUC of MSIsensor-RNA with inconsistent training and testing samples**

| **Train data** | **CRC** | **STAD** | **UCEC** | **All** | **Mean** |
| --- | --- | --- | --- | --- | --- |
| CRC | 1.0000 | 0.9183 | 0.7705 | 0.8808 | 0.8924 |
| STAD | 0.9495 | 1.0000 | 0.9068 | 0.8246 | 0.9202 |
| UCEC | 0.8846 | 0.9904 | 0.9932 | 0.9532 | 0.9553 |
| Three type merged | 0.9880 | 1.0000 | 0.9523 | 0.9761 | 0.9791 |

*Note*: CRC colorectal cancer; STAD, stomach adenocarcinoma; UCEC, uterine corpus endometrial carcinoma; AUC, area under the receiver operating characteristic curve.
