# Supplementary material for: MSIsensor-RNA: Microsatellite Instability Detection for Bulk and Single-cell Gene Expression Data
Source: Genomics Proteomics Bioinformatics. 2024 Jan 10;22(3):qzae004. doi: 10.1093/gpbjnl/qzae004 (PMC12016039; doi:10.1093/gpbjnl/qzae004)
Supplement: qzae004_Supplementary_Data [file qzae004_supplementary_data.zip › Table S17-done.docx]

**Table S17 Performance of train models for cancer with low-frequency MSI**

| **Model** | **No of sample** | **AUC** | **F1-score** | **Accuracy** | **Sensitivity** | **Specificity** | **Precision** |
| --- | --- | --- | --- | --- | --- | --- | --- |
| CRC | 3442 | 0.6113 | 0.0169 | 0.6630 | 0.6645 | 0.4348 | 0.0086 |
| STAD | 3442 | 0.6535 | 0.0000 | 0.9933 | 1.0000 | 0.0000 | NA |
| UCEC | 3442 | 0.6111 | 0.0325 | 0.9654 | 0.9713 | 0.0870 | 0.0200 |
| Three type merged | 3442 | 0.6239 | 0.0690 | 0.9686 | 0.9740 | 0.1739 | 0.0430 |
