# Supplementary material for: MSIsensor-RNA: Microsatellite Instability Detection for Bulk and Single-cell Gene Expression Data
Source: Genomics Proteomics Bioinformatics. 2024 Jan 10;22(3):qzae004. doi: 10.1093/gpbjnl/qzae004 (PMC12016039; doi:10.1093/gpbjnl/qzae004)
Supplement: qzae004_Supplementary_Data [file qzae004_supplementary_data.zip › Table S18-done.docx]

**Table S18 Performance of MSIsensor-RNA for cancer with low-frequency MSI by 5-fold cross-validation**

| **ID** | **Train or test?** | **No. of sample** | **AUC** | **F1-score** | **Accuracy** | **Sensitivity** | **Specificity** | **Precision** |
| --- | --- | --- | --- | --- | --- | --- | --- | --- |
| 0_train | train | 2753 | 1.0000 | 0.8780 | 0.9982 | 0.9982 | 1.0000 | 0.7826 |
| 0_test | test | 689 | 0.5512 | 0.0000 | 0.9869 | 0.9942 | 0.0000 | 0.0000 |
| 1_train | train | 2753 | 1.0000 | 0.8372 | 0.9975 | 0.9974 | 1.0000 | 0.7200 |
| 1_test | test | 689 | 0.6018 | 0.0000 | 0.9884 | 0.9956 | 0.0000 | 0.0000 |
| 2_train | train | 2753 | 1.0000 | 0.9474 | 0.9993 | 0.9993 | 1.0000 | 0.9000 |
| 2_test | test | 689 | 0.5323 | 0.0000 | 0.9898 | 0.9971 | 0.0000 | 0.0000 |
| 3_train | train | 2753 | 1.0000 | 0.7660 | 0.9960 | 0.9960 | 1.0000 | 0.6207 |
| 3_test | test | 689 | 0.5772 | 0.0000 | 0.9898 | 0.9971 | 0.0000 | 0.0000 |
| 4_train | train | 2753 | 1.0000 | 0.9730 | 0.9996 | 0.9996 | 1.0000 | 0.9474 |
| 4_test | test | 689 | 0.4863 | 0.0000 | 0.9898 | 0.9971 | 0.0000 | 0.0000 |

*Note*: AUC, the area under the receiver operating characteristic curve.
