# Supplementary material for: MSIsensor-RNA: Microsatellite Instability Detection for Bulk and Single-cell Gene Expression Data
Source: Genomics Proteomics Bioinformatics. 2024 Jan 10;22(3):qzae004. doi: 10.1093/gpbjnl/qzae004 (PMC12016039; doi:10.1093/gpbjnl/qzae004)
Supplement: qzae004_Supplementary_Data [file qzae004_supplementary_data.zip › Table S20-done.docx]

**Table S20 Summary of MSI-H training samples for STAD in Table S19**

| **Cancer stage** | **No. of samples** | **Ratio of samples** |
| --- | --- | --- |
| I | 10 | 0.16 |
| II | 18 | 0.29 |
| III | 22 | 0.35 |
| IV | 7 | 0.11 |
| NA | 5 | 0.08 |
