# Supplementary material for: MSIsensor-RNA: Microsatellite Instability Detection for Bulk and Single-cell Gene Expression Data
Source: Genomics Proteomics Bioinformatics. 2024 Jan 10;22(3):qzae004. doi: 10.1093/gpbjnl/qzae004 (PMC12016039; doi:10.1093/gpbjnl/qzae004)
Supplement: qzae004_Supplementary_Data [file qzae004_supplementary_data.zip › Figure S1.pptx]

## Slide 1
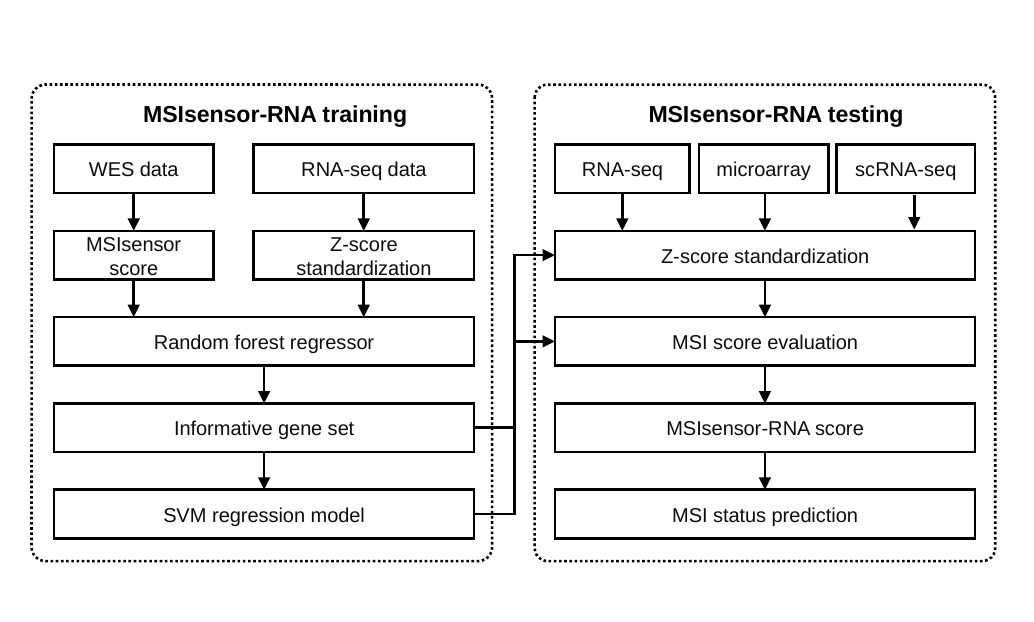

MSIsensor-RNA training
MSIsensor-RNA testing
WES data
RNA-seq data
RNA-seq
microarray
scRNA-seq
MSIsensor score
Z-score standardization
Z-score standardization
Random forest regressor
MSI score evaluation
Informative gene set
MSIsensor-RNA score
SVM regression model
MSI status prediction
