# Supplementary material for: MSIsensor-RNA: Microsatellite Instability Detection for Bulk and Single-cell Gene Expression Data
Source: Genomics Proteomics Bioinformatics. 2024 Jan 10;22(3):qzae004. doi: 10.1093/gpbjnl/qzae004 (PMC12016039; doi:10.1093/gpbjnl/qzae004)
Supplement: qzae004_Supplementary_Data [file qzae004_supplementary_data.zip › Figure S2.pptx]

## Slide 1
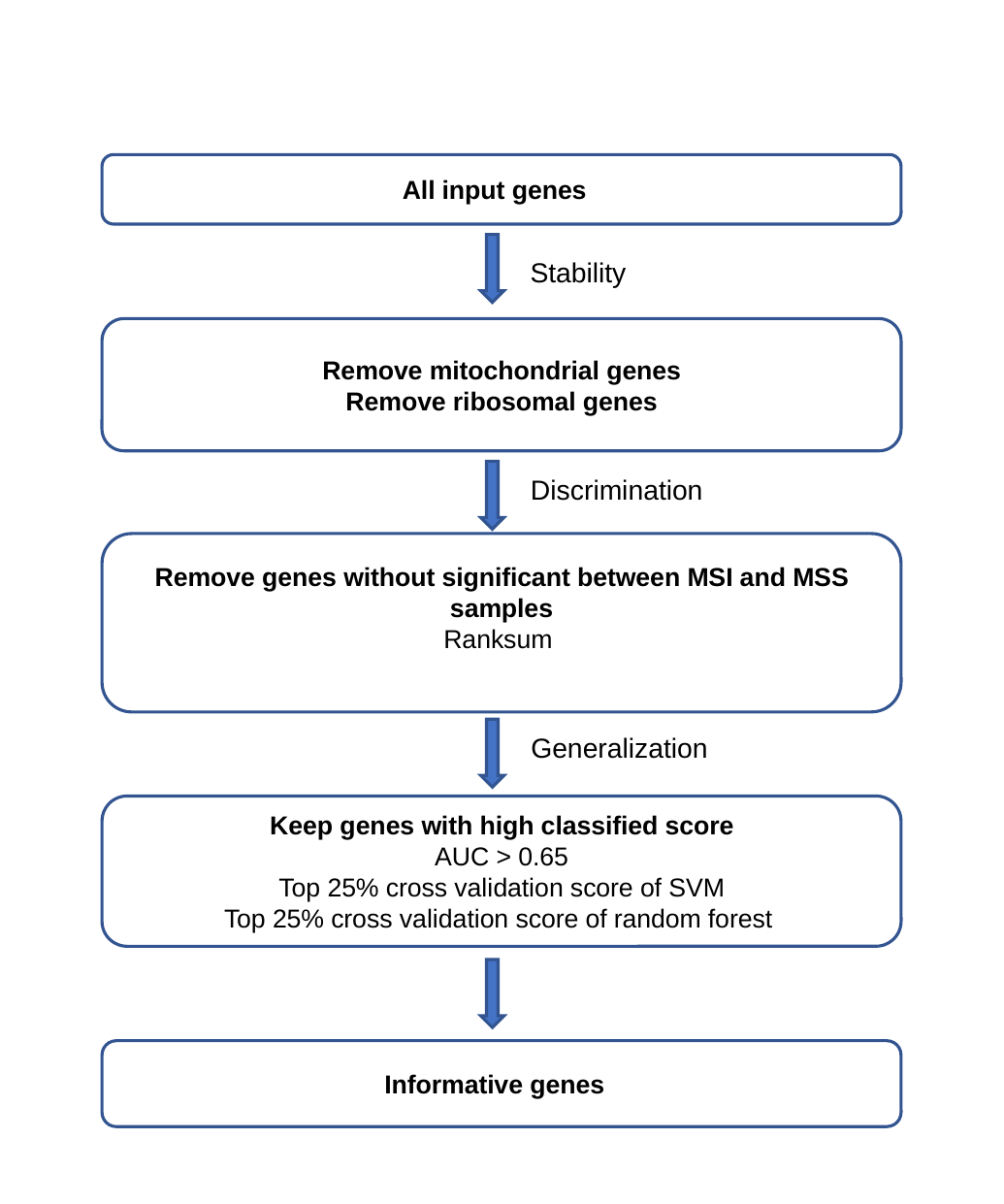

All input genes
Stability
Remove mitochondrial genes
Remove ribosomal genes
Discrimination
Generalization
Keep genes with high classified score
AUC > 0.65
 Top 25% cross validation score of SVM
Top 25% cross validation score of random forest
Informative genes
