# Supplementary material for: MSIsensor-RNA: Microsatellite Instability Detection for Bulk and Single-cell Gene Expression Data
Source: Genomics Proteomics Bioinformatics. 2024 Jan 10;22(3):qzae004. doi: 10.1093/gpbjnl/qzae004 (PMC12016039; doi:10.1093/gpbjnl/qzae004)
Supplement: qzae004_Supplementary_Data [file qzae004_supplementary_data.zip › Figure S4.pptx]

## Slide 1
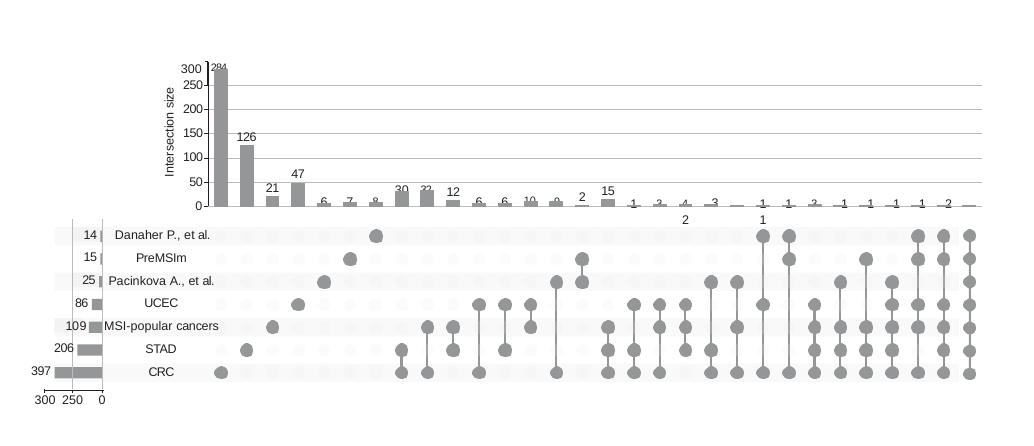

300 284
250
200
150
100
50
0
Intersection size
126
47
21
30 32
15
12
6 6 10 9
6 7 8
2
4 3 2
1 3
1 1 3 1 1 1 1 2 1
Danaher P., et al.
14
15
PreMSIm
Pacinkova A., et al.
25
86
UCEC
9 MSI-popular cancers
10
206
STAD
397
CRC
300 250 0
