# Supplementary figures and images for: MSIsensor-RNA: Microsatellite Instability Detection for Bulk and Single-cell Gene Expression Data
Source: Genomics Proteomics Bioinformatics. 2024 Jan 10;22(3):qzae004. doi: 10.1093/gpbjnl/qzae004 (PMC12016039; doi:10.1093/gpbjnl/qzae004)

## Slide 1
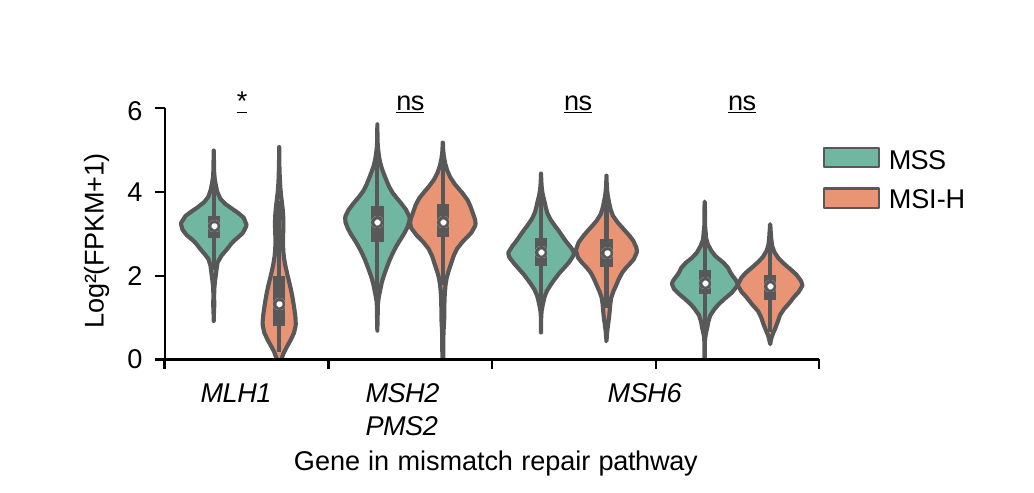

*
	ns
	ns
	ns
6
MSS MSI-H
Log²(FPKM+1)
4
2
0
MSH2	MSH6	PMS2
Gene in mismatch repair pathway
MLH1

Supplement: qzae004_Supplementary_Data [file qzae004_supplementary_data.zip › Figure S5.pptx]

## Slide 1
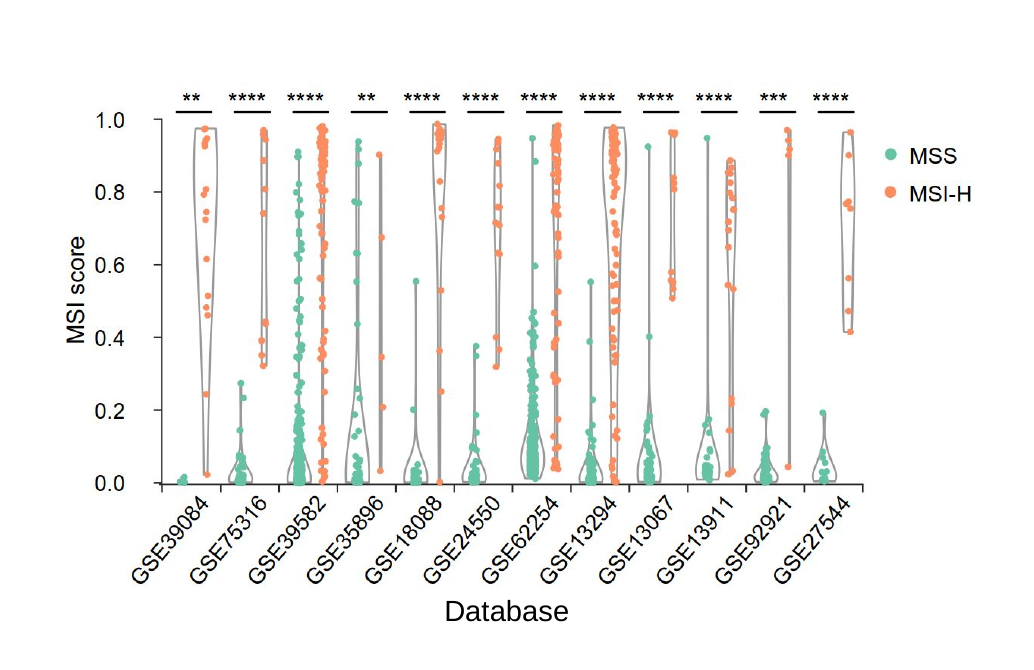

Database

Supplement: qzae004_Supplementary_Data [file qzae004_supplementary_data.zip › Figure S6.pptx]

## Slide 1
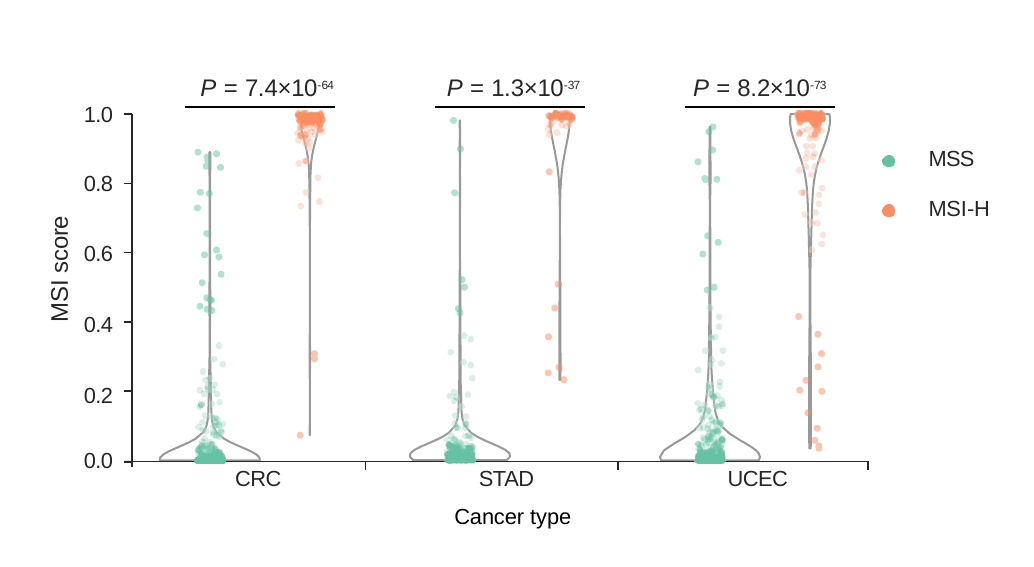

P = 7.4×10-64
P = 1.3×10-37
P = 8.2×10-73
1.0
MSS
0.8
MSI-H
MSI score
0.6
0.4
0.2
0.0
CRC
STAD
UCEC
Cancer type

Supplement: qzae004_Supplementary_Data [file qzae004_supplementary_data.zip › Figure S8.pptx]

## Slide 1
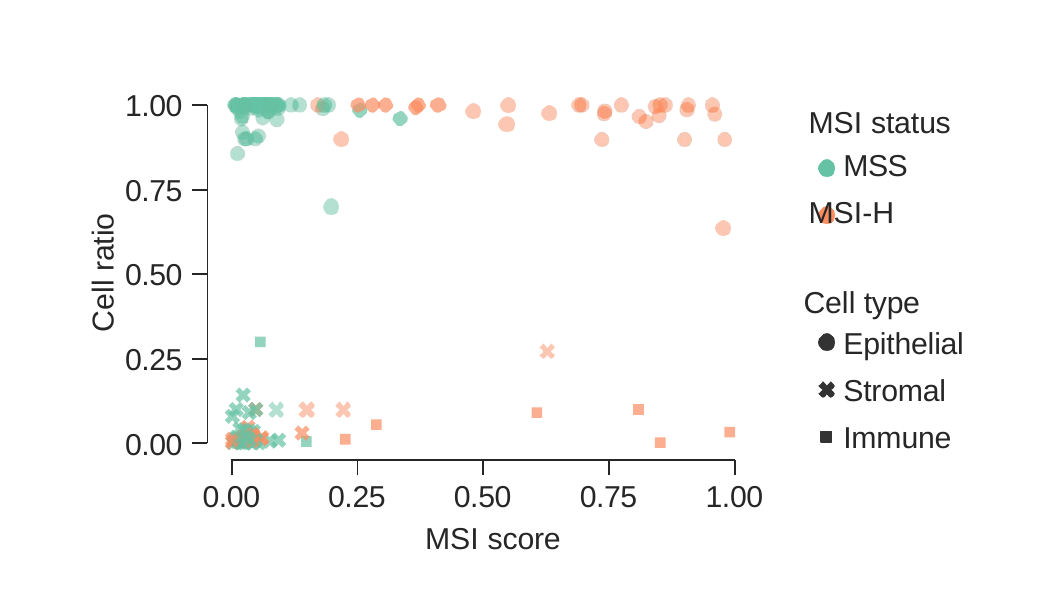

1.00
MSI status MSS
MSI-H
0.75
Cell ratio
0.50
Cell type
Epithelial
Stromal Immune
0.25
0.00
0.50
MSI score
0.00
0.25
0.75
1.00

Supplement: qzae004_Supplementary_Data [file qzae004_supplementary_data.zip › Figure S11.pptx]

## Slide 1
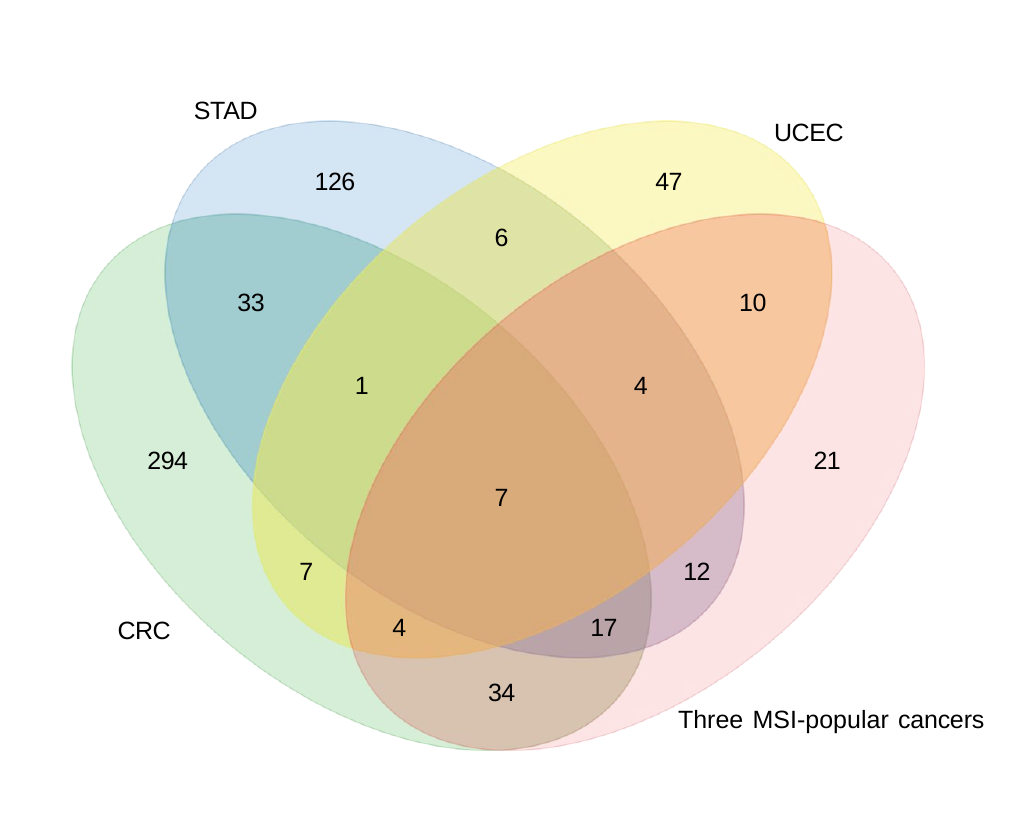

STAD
UCEC
126
47
6
33
10
1
4
294
21
7
7
12
4
17
CRC
34
Three MSI-popular cancers

Supplement: qzae004_Supplementary_Data [file qzae004_supplementary_data.zip › Figure S3.pptx]
